# Editorial: Advancing Genomics for Rare Disease Diagnosis and Therapy Development

**DOI:** 10.3389/fphar.2020.598889

**Published:** 2020-09-25

**Authors:** Zhichao Liu, Ruth Roberts, Tieliu Shi, Mike Mikailov, Weida Tong

**Affiliations:** ^1^Division of Bioinformatics and Biostatistics, National Center for Toxicological Research, US Food and Drug Administration, Jefferson, AR, United States; ^2^Department of Drug Safety, ApconiX, Alderley Edge, United Kingdom; ^3^Department of Biosciences, University of Birmingham, Birmingham, United Kingdom; ^4^Office of Science and Engineering Labs, Center for Devices and Radiological Health, US Food and Drug Administration, Silver Spring, MD, United States

**Keywords:** rare dieases, NGS, genomics, diagnosis, drug discovery

Rare diseases affect only a small percentage of the population and are often chronic and potentially life-threatening. There are more than 7,000 known rare diseases, and yet fewer than 700 approved treatment options are available. Progress made with the use of emerging technologies such as next-generation sequencing (NGS) and bioengineering holds great promise in advancing rare disease diagnosis and therapy development (Liu et al., 2019). This Research Topic brings together a multi-faceted approach to improve rare disease diagnosis and treatment development using genomic technologies, including (1) causal genetic variant identification (2) experimental designs for genetic testing studies; (3) population-specific genetic testing; (4) genotype-phenotype association; (5) enhanced diagnostic power; (6) retrospective and prospective studies; (7) reproducibility of NGS-based genetic testing; and (8) data management and bioinformatics pipeline development.

Identification of disease-causing variants is essential for improving our understanding of disease history and accelerating diagnostic biomarker discovery for rare diseases. NGS technology provides unprecedented speed and resolution to reveal individual genetic makeup, underpinning the identification of rare disease causal variants. Girotto et al. employed targeted sequencing to screen a large cohort of 464 Italian patients to prioritize genetic variants related to age-related hearing loss (ARHL). They found unreported genetic variants in SLC9A3R1 and further confirmed the finding using an in-vivo zebrafish knock-in model. Gallego-Martinez et al. conducted a targeted sequencing analysis of 890 Sporadic Ménière’s disease (MD) patients to identify the rare missense variants associated with hearing loss-related genes. These studies helped design a “fit-for-purpose” gene panel to diagnose MD. Liu et al. used targeted sequencing to find a compound heterozygous inherited deletion in the CHAT gene in a Chinese patient with severe congenital myasthenic syndrome with episodic apnea (CMS-EA). Besides targeted sequencing, there are increasing applications of whole genome sequencing/whole exome sequencing (WGS/WES) to detect complex genetic variants and provide complete genetic information in support of rare disease diagnosis. Yan et al. identified novel neonatal variants associated with Carbamoyl phosphate synthetase I deficiency (CPS1D) using WES, and further verified their findings based on a comprehensive literature survey; this expanded our knowledge of the genetic variants of the CPS1 gene and associated phenotypes.

NGS that deploys trio-based and proband sampling design allows for more sensitive detection of *de novo* mutations that are present only in children, providing useful information on variants in recessive or imprinted disorders by inheritance. Kausar et al. carried out WES analysis on three spondyloocular syndrome (SOS) patients in a consanguineous Pakistani family and found a novel homozygous frameshift variant in XYLT2. Sanger sequencing was further used to confirm the findings and to reveal an autosomal recessive inheritance pattern. Shahid et al. reported a novel, homozygous FERMT3 nonsense mutation (c.286C > T, p.Q96_) in the proband of an infant in a Pakistani family by using targeted sequencing. Novel causal variants may also facilitate the prenatal diagnosis of Leukocyte adhesion deficiency-III (LAD3). Zhang et al. associated the genetic variants (i.e., FLT4: c.3075G>A) identified from a Chinese trio Milroy disease (MD) family to different histological changes, providing more insight into the pathogenic impact of FLT4 mutations. Laugel-Haushalter et al. employed WES to detect SLC10A7 mutations in Amelogenesis imperfecta (AI) in an affected daughter from a consanguineous family, indicating a diversity of phenotypes associated with the mutations. Ren et al. identified three novel mutations (c.1551+1insTGAT in TPP1, c.244G>T in CLN6, c.554-5A>G in MFSD8) in genes associated with Late-Infantile Neuronal Ceroid Lipofuscinosis (LINCLs). The authors conducted third generation sequencing (TGS) with downstream pathological assessment and functional analysis on four late-infantile NCL siblings with similar phenotypic symptoms and found mutations located in different genes. Lu et al. used Sanger sequencing and WES to investigate a Chinese family in which two siblings were affected by the infantile form of primary hyperoxaluria type 1 (PH1). Two novel missense mutations were identified for the infantile form of PH1 by WES. Furthermore, the authors found that the same AGXT genotype caused the same infantile form of PH1 within the family.

A better understanding of allelic frequency across different populations is key to implementing “fit-for-purpose” diagnostic tools in rare diseases. Zhytnik et al. presented a Sanger sequencing analysis on 94 Ukrainian Osteogenesis imperfecta (OI) families. They identified 27 novel COL1A1/2 pathogenic variants, indicating that the spectrum of OI genotypes may differ between populations. Yue et al. conducted a case-control population study to screen the allele frequency of SERPINC1 variant rs2227589 in the Chinese population using the Sequenom assay. The study suggested that variant rs2227589 was associated with an increased risk of antithrombin deficiency in pulmonary embolism (PTE). Fu et al. carried out a meta-analysis by collecting 117 HMBS gene mutations from acute intermittent porphyria (AIP) patients and evaluated mutational impact on corresponding protein structures and functions. The authors found population disparities within 23 genetic variants across eight different ethnic groups, providing important information on population-based diagnostic biomarker development for AIP.

Although NGS can provide more information on the detailed genetic makeup of rare disease patients, it remains challenging to precisely identify pathogenic variants with potential clinical applications. Several studies reviewed in this Research Topic explored different phenotypic anchoring strategies to establish the genotype-phenotype association in rare diseases. Hong et al. screened 540 patients from 187 unrelated Chinese Von Hippel–Lindau (VHL) families for 19 frequent VHL mutations, looking for associations between allelic frequency and clinical phenotypes. Furthermore, a Kaplan–Meier survival analysis was carried out to link allelic frequency with onset age. Lin et al. conducted a phenotypic association study, and found that Sodium Taurocholate Cotransporting Polypeptide (NTCP) deficiency could be covered up by citrin deficiency during early infancy for neonatal patients with the pathogenic variant c.800C > T (p.Ser267Phe) in gene SLC10A1. Feng et al. investigated clinical manifestations and genetic abnormalities in children with Familial hemophagocytic lymphohistiocytosis Type 2 (FHL2) using WES and enhanced magnetic resonance imaging (MRI). Lv et al. reported a case study of a mutant c.2185C > T in the RPS6KA3 gene associated with Coffin-Lowry syndrome (CLS), identified by targeted sequencing with magnetic resonance imaging (MRI). Further, they discussed the efficacy and safety of the application of growth hormone analogs in patients with CLS. Song et al. investigated the phenotype of patients with NR5A1 gene mutations in 30 Chinese patients (11 boys and 19 girls). No gender difference was identified regarding associated phenotypes due to genetic variants of NR5A1, but NR5A1 genetic variants were linked to different clinical manifestations. Zhao et al. conducted a retrospective multicenter study of 141 patients with 5a-reductase type 2 deficiency (5aRD). They investigated growth patterns and their association with clinical parameters such as luteinizing hormone (LH).

A combination of NGS and other advanced analytical technologies will enhance diagnostic power and the identification of disease-causing variants. Zhou et al. integrated NGS and tandem mass spectrometry (MS/MS) to screen 236,368 newborns for Primary carnitine deficiency (PCD) to precisely diagnose PCD. Wang D. et al. proposed a hybrid approach by combining multiplex ligation-dependent probe amplification (MLPA) and NGS to improve the precise identification of genetic variants in 70 Chinese families with suspected Muscular dystrophy (MD) probands.

Retrospective and prospective study using meta-analysis of reported genetic variants, especially for rare diseases, is an effective way to generate hypotheses and enhance understanding of underlying molecular mechanisms. Shi et al. implemented a meta-analysis to categorize the clinical phenotypes and genotypes based on variant types for 155 OI patients collected from the literature. The authors reported that three phenotypes (bone deformity, DI, walking with assistance) were enriched in two variation types (the Gly-substitution missense; and groups of frameshift, nonsense, and splicing variations). Li et al. conducted a prospective study to characterize the phenotypic, genetic, and electroencephalographic features of children with DNM1 mutation-related epileptic encephalopathy. The authors suggested that patients carrying pathogenic variants in the GTPase or middle domains present different epileptic encephalopathy and neurodevelopmental symptoms. Shahryari et al. reviewed 20 approved human gene and cell-based gene therapy products with great promise in treating devastating rare diseases and cancers. The pros and cons of these 20 gene therapy products were compared, and potential solutions for further improvement were provided.

Reproducible NGS-based genetic testing is vital for a successful clinical diagnosis of rare diseases. Liang et al. carried out a comparative analysis of a *de novo* variant calling with the state-of-the-art bioinformatics pipeline. The authors found a suboptimal concordance among the different calling algorithms and proposed a filtering strategy to improve the reproducibility of *de novo* variant identification.

Knowledge management and bioinformatics software development is essential for filtering and prioritizing clinically relevant genetic variants in rare diseases. Wang X. et al. introduced “Mingjian” software to improve genetic variant annotations. “Mingjian” software is a self-updating genetic disease computer-supported diagnostic system that integrates variant annotation databases, including HPO, OMIM, HGMD, with sophisticated statistical measures. Clinical utility of the software was verified with WES by using genetic variants detected from 30 neurological patients. Zuo et al. explored novel genetic elements such as piwi-interacting RNAs (piRNAs) for their potential as prognostic biomarkers in the recurrence of prostate cancer using network analysis. Huang et al. created NCATS BioPlanet, a comprehensive, integrated pathway resource consisting of a universe of 1,658 human pathways sourced from publicly available, manually curated sources. It is a promising tool to facilitate research in systems biology, toxicology, and chemical genomics.

The power and promise of genomics for the diagnosis of rare diseases are exemplified in this special issue. However, many essential aspects of genomics in rare diseases were not included in this review of a small collection of articles. For example, drug repositioning for rare disease treatment development, standardization of rare disease terminology for clinical application, and novel NGS technologies are also of significant importance in widening the horizon of genomics application. We hope this Research Topic will trigger a broader interest and heightened community discussion toward the advancement of rare disease diagnosis and treatment development.

## Author Contributions

ZL wrote the first draft of the editorial. WT, RR, MM, and TS revised the editorial.

## Disclaimer

The views presented in this article do not necessarily reflect current or future opinions or policies of the US Food and Drug Administration. Any mention of commercial products is for clarification and not intended as an endorsement.

## Conflict of Interest

RR is co-founder and co-director of ApconiX, an integrated toxicology and ion channel company that provides expert advice on non-clinical aspects of drug discovery and drug development to academia, industry, and not-for-profit organizations.

The remaining authors declare that the research was conducted in the absence of any commercial or financial relationships that could be construed as a potential conflict of interest.
